# Lake morphology and meteorological conditions impact stratification of saline lakes in the Atacama Desert

**DOI:** 10.1371/journal.pone.0321759

**Published:** 2025-05-05

**Authors:** Tianshu Kong, R. Pamela Reid, Erica P. Suosaari, Daniela Maizel, Luis R. Daza, Alvaro T. Palma, Amanda M. Oehlert

**Affiliations:** 1 Department of Marine Geosciences, Rosenstiel School of Marine, Atmospheric, and Earth Science, University of Miami, Miami, Florida, United States of America; 2 Department of Mineral Sciences, National Museum of Natural History, Smithsonian Institution, Washington, Columbia, United States of America; 3 Department of Ocean Sciences, Rosenstiel School of Marine, Atmospheric, and Earth Science, University of Miami, Miami, Florida, United States of America,; 4 Fisioaqua, Las Condes, Santiago, Chile; Centro de Astrobiologia, SPAIN

## Abstract

Saline lakes exist in various morphologies within salar environments, occurring as ephemeral to persistent bodies of water despite evaporative conditions. Salar environments are often characterized by strong diurnal fluctuations in temperature, UV irradiation, and wind speed, however, the extent to which these meteorological conditions impact saline lakes with different morphological characteristics has yet to be investigated. Here, we evaluate the impacts of diurnal changes in wind speed and wind direction on lake water temperature, electrical conductivity, alkalinity, and stable isotope ratios of hydrogen (δ^2^H), oxygen (δ^18^O), and sulfur (δ^34^S_SO4_) in two Na-Cl saline lakes in the Salar de Llamara (Atacama Desert, Northern Chile) with different morphologies. Results indicate that water masses in the relatively deeper (~ 0.6 m) steep-sided lake with crystalline lake bottom remained stratified despite the nearly order of magnitude increase in diurnal wind speed, while waters in the relatively shallow (< 0.2 m), gently-sloping lake colonized by microbial mats were well-mixed. Conditions in the shallow, gently-sloping lake were heterogeneous, with diurnal variations approximating 15.6% and 23.9% of known seasonal ranges in temperature and electrical conductivity, respectively. Consequently, the chemistry of shallow, gently-sloping saline lake environments is dynamic on diurnal time scales, indicating that resident microbial communities tolerate a greater range in environmental conditions than previously appreciated. Results suggest that the impacts of diurnal changes in meteorological conditions on saline lake stratification depend on lake morphology, an observation with implications for patterns of mineral deposition in salar environments.

## Introduction

Northern Chile is home to more than 100 salars, spanning a total of more than 7,252 square kilometers [1], and home to more than 16 Andean microbial ecosystems [2]. Some of these salars, like the Chile’s Salar de Atacama or Bolivia’s Salar de Uyuni, host economically significant quantities of lithium, boron, sodium chloride, iodine, potassium, and magnesium, which are valuable commodities [3], including materials used for batteries [4] and medicinal uses [5]. Other salars are home to a variety of Andean microbial ecosystems, ranging from microbial mats to endoevaporitic ecosystems, stromatolites, thrombolites, and oncolites [6]. Living at the edge of habitability, these microbial ecosystems persist in polyextreme environments [sensu 7], where multiple environmental parameters exhibit extreme ranges, including high UV irradiation, diurnal temperature swings, aridity, and in some cases high salinity and/or arsenic contents [2], water activity [8], chaotropicity [9], or acidity [10].

The diverse geological expression in these salar environments includes as a range of minerals, from evaporites like gypsum, halite, eugsterite, thenardite, anhydrite, and sylvite, to carbonate minerals like aragonite and calcite, as well as clays [2,11,12]. Understanding the role of water or brine chemistry on the formation and distribution of minerals within salar environments can aid in the interpretation of their depositional settings in the ancient geological record. Bąbel (2004) evolved previous models of evaporite deposition and mineralogy [13–15, among others] to provide a detailed conceptual framework for gypsum morphologies, including microbial gypsum and selenite, as well as other evaporite minerals like halite and their deposition in ancient basins. One of the key mechanisms identified in this conceptual model as a driver of mineral precipitation in evaporite basins was vertical stratification and periodic mixing [16], a phenomenon that is beginning to be described in studies of the saline lake systems within some of salars of Northern Chile [12,17,18].

Stratification is an important feature that can affect the style, type, and rate of gypsum deposition. Consequently, understanding the frequency of brine column mixing is fundamental to studies of mineral deposition in brine pools and lakes [15]. Meteorological conditions are known to establish both stratification and currents in lakes, and influential parameters include air temperature, wind speed, and solar radiation [19]. Turbulent mixing and heat exchange can exert strong influences on the hydrology, geochemistry, biological activity, and productivity of lake systems [19]. Long-lived and stable density stratification can decouple bottom brines from atmospheric connection, allowing the bottom brines to chemically evolve through time due to limited mixing with the upper water mass [16,20], while periodic mixing can regenerate depleted supplies of nutrients and ions (*i.e.,* calcite, iron, and manganese) as well as oxygen [19]. Chemical stratification may also promote variations in mineral saturation state. Such stratification has been interpreted to influence the deposition of extensive selenite pan deposition in stratified brines [13,14,16], as well as mineral dissolution in some instances [21], which can ultimately change the composition of the lake waters as a result [22].

**Fig 1:**
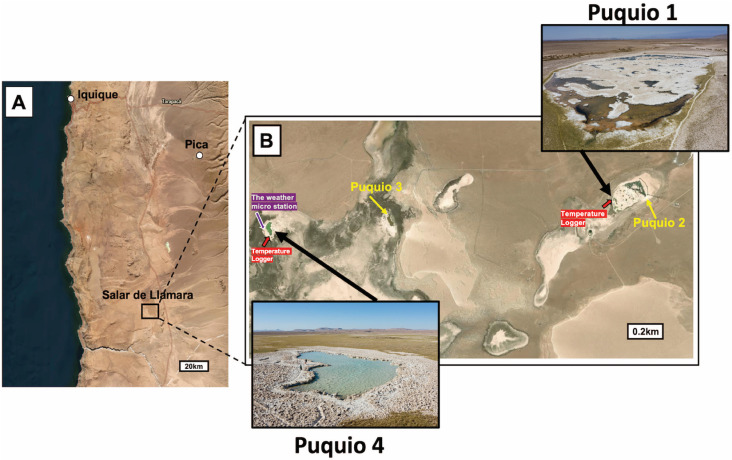
Study location. (A) Map of the Salar de Llamara, located in the Atacama Desert, northern Chile. (B) Location map of the Puquios; Puquios 1 (area: 1760 m^2^, fetch: 17 m, maximum depth: 50 cm) and Puquio 4 (area: 1485 m^2^, fetch: 47 m, maximum depth: 80 cm) are the focus of this study. The weather micro station (purple symbol) and temperature loggers (red symbols) are shown in in Fig 1B. Background maps (A) and (B) were created using ArcGIS Pro.

The Salar de Llamara saline lake system (‘Puquios’, 21°23’ S, 69°37’ W) is an ideal study site to investigate environmental controls on brine stratification and diurnal mixing (Fig 1). The Puquios are a system of relatively shallow saline lakes characterized by distinct lake morphologies, degrees of stratification, lake bottom types *(i.e.,* crystalline vs. microbial), and degree of microbial influence on mineral deposition [12,17,18,23]. Recent work suggests the waters and brines in Puquios 1 and 2 are vertically stratified across a variety of scales with respect to a range of chemical parameters, including temperature, electrical conductivity, pH, and total dissolved solids [12,17,18]. In many of these studies, bottom measurements of geochemical parameters, such as the concentration of total dissolved solids or electrical conductivity, typically exceed those of the surface measurements during both austral fall and spring [12,17]. Reid et al. [18] expanded upon the spatial analysis of Otálora et al. [12] and included measurements in the main lakes and peripheral ponds of Puquios 3 and 4. Electrical conductivity (EC), a proxy for salinity, was found to vary substantially (and predictably) across the depositional environment, with the highest EC values observed in the main saline lakes of Puquio 2 and 4, and the lowest EC values observed in the main lakes of Puquios 1 and 3 and their peripheral ponds [17]. Water column stratification, calculated following the definition presented by Bąbel [16], was typically found to be normally stratified in the main lakes of Puquios 1, 2, and 4, with the exception of Puquio 3, which was nearly homogeneous at the time of measurement [17]. Stratification of in situ measurements of EC, dissolved oxygen concentrations (DO), total dissolved solids (TDS) in the Salar de Llamara were predominantly observed in the saline lakes that are relatively deep (> 30 cm) and steep sided (Puquios 2 and 4), resulting in crystalline substrates on the lake bottom [18]. Conversely, shallow, gently sloping lakes were more often characterized by high degrees of lateral variability in brine chemistry and predominantly contained microbial substrates [18]. Finally, a recent study has suggested that mushroom-shaped gypsum structures in Puquio 2 were shaped by stratified waters characterized by variations in gypsum saturation state [21]. In concert, these studies demonstrate that lake stratification is increasingly recognized as an important influence on the chemistry and sedimentology of saline lake settings in the Atacama Desert.

Environmental conditions, such as wind speed and wind direction, are known to vary substantially and predictably throughout the diurnal cycle in the Atacama Desert [24]. Previous work has shown that wind-driven mixing plays a role in determining the depth of the brine interfaces [25], but the influence of variations in wind speed and its impact on lake chemistry and water column stratification have yet to be investigated in the Atacama Desert. Motivated by this knowledge gap, the objectives of the present study were to collect contemporaneous measurements of surface and bottom water chemistry and temperature, as well as measurements of environmental conditions, like wind speed and direction, to assess the role of saline lake architecture in maintaining vertical stratification. We hypothesize that the relatively deeper, steeper-sided Puquio 4 (21°270’S, 69°636’W) will remain stratified throughout a diurnal cycle, while lake waters in the shallow, gently sloping Puquio 1 (21°268’S, 69°618’W) will be vertically mixed by more intense wind speeds that occur in the afternoon. To test this hypothesis, we evaluated *in situ* meteorological data, water temperature measurements collected by loggers installed at two water depths in each lake, and discrete samples collected in both the surface and bottom waters in the morning and afternoon of November 12^th^ and 13^th^, 2019.

## Geological setting

The Puquios in the Salar de Llamara are located in one of the main endorheic basins in the Pampa del Tamarugal within the Central Depression [26–28]. The Pampa del Tamarugal is a catchment for sediments and water from the Arcas Alluvial Fan [26,29] and contains thick sequences of lacustrine-evaporitic sediments deposited since the Miocene [28,30]. The region is extremely arid, with rainfall estimated to be less than one millimeter per year in the core of the Atacama Desert [31], with high rates of evaporation that exceed rainfall [32]. The Salar de Llamara itself is a remnant of a massive lake known as the Gran Lago Soledad [33]that was thought to have reached depths of nearly 80 m [1]. The region experienced rapid desertification between 8,000 and 3,000 years ago, generating many salars that can be observed in a north-south transect between the Coastal Cordillera and the Andes Mountain Chains [15,27]. Saline lakes can be found in several of these salars, including the Puquios in the Salar de Llamara, one of several modern gypsum-depositing environments in the region [12,17,18,23,34–37]. These saline lakes, and several others in the region, are hydrologically supported by a combination of summer monsoons (Invierno Altiplánico) in the High Cordillera [38–40], along with minor occurrences of coastal dripping fog [41,42].

The Puquios are variably sized depressions filled with groundwater, characterized by a complex interplay of physical, chemical, geological, and biological processes [12,17,18,23]. Within this salar, there are four main lakes (Puquios 1, 2, 3, and 4) and over 400 smaller peripheral ponds located within an area of less than 5 km^2^ (Fig 1). Each saline lake exhibits variability in electrical conductivity, benthic and planktonic biota, and mineral deposition styles, and multi-disciplinary research has documented heterogeneity in the chemistry, microbiology, sedimentology, and mineralogy in the Puquios [12,17,18,23,34–36,43–45]. In terms of chemical composition, previous studies have outlined a clear impact of evaporative concentration on the dominantly Na-Cl brines in the Salar de Llamara [12,17,34,46]. Density stratification and lateral gradients in salinity, temperature, pH, oxidation/reduction potential, and dissolved oxygen were found to persist across seasons across samples collected in the Transition Zone and Puquio 2 from the Salar de Llamara [12]. Past studies have demonstrated that Puquio 2 has significantly higher concentrations of most major ions and more pronounced stratification compared to Puquio 1[12,17,46], and Puquio 4 has more concentration ionic composition than Puquio 3 [17,18]. With respect to the dominant ions, Na^+^ and Cl^-^, the ionic composition of Puquio 4 is ~ 2 × that of Puquio 3, and the ionic composition of Puquio 2 is concentrated by a factor of ~ 8–16 compared to Puquio 1[17]. Variation in the minor element concentrations, particularly manganese and strontium, in the lake waters were interpreted to reflect differences in subsurface lithology and/or water sources, which produced observable differences in the chemistry of minor mineral assemblages [17].

In terms of lake morphology, Puquio 1 is a large shallow lake with clear water, covering an area of 1760 m^2^ and reaching depths of up to 40 cm [17]. The main saline lake of Puquio 1 is characterized by relatively low average electrical conductivity (average ~ 30 mS/cm in surface and bottom waters; [17]). In Puquio 1, the lake bottom substrate consists of an unlithified, flocculant microbial mat containing irregular precipitates of gypsum, aragonite, and clay [17,18,23]. Puquio 4 spans 1485 m^2^ with depths approximately 2 × those of Puquio 1 (up to 80 cm; [17]) and vibrant turquoise waters (Fig 1). Although similar in size to Puquio 1, Puquio 4 is relatively deeper, and is characterized by a steeper gradient from the margin to the lake bottom, with generally high average electrical conductivity (180.7 mS/cm in surface and 179.3 mS/cm in the bottom; [17]). The lake bottom substrate in Puquio 4 is characterized by crystalline bottom types that lack significant accumulations of microbial biofilms [17,18,23]. Calculations of slope grade based on subaqueous transects from bathymetric maps of Puquios 1 and 4 [17,47] suggest significantly different average slope grades for Puquio 1 (1.6 ± 0.4%; *n* = 4) and Puquio 4 (4.5 ± 1.5%; *n* = 4, *p* = 0.03, two-tailed t-test assuming unequal variance). Field observations suggest these estimates of grade are conservative and represent the percent slope in the subaqueous environment; the subaerial sidewalls of Puquio 4 immediately adjacent to the air-water interface are nearly vertical, while the low percent slope in the subaqueous portions of Puquio 1 persists across the transition from subaqueous-subaerial depositional environments (Fig 1).

## Methods

Field work in the Salar de Llamara was conducted with permission from SUBPESCA in Chile (permiso de pesca de investigación, RES EX No. 3477 14-11-2019). Meteorological conditions were measured using a weather micro station (HOBO H21-USB Micro Station Data Logger, Onset, Cape Cod, Massachusetts, USA) installed approximately 120 m NW-W of Puquio 4 (Latitude: 21°270’S, Longitude: 69°636’W, elevation: ~ 2,500 feet). The weather micro station was equipped with a RM Young Marine Wind Monitor for wind speed, gust speed & direction, a Photosynthetically Active Radiation (PAR) smart sensor and a Temperature/Relative Humidity smart sensor installed ~ 3 m above the ground. Measurement parameters were recorded every 10 minutes with 1 minute observation periods averaged for each measurement. Using the prevailing wind direction measured by the weather micro station, fetch was calculated for both Puquio 1 and Puquio 4 by measuring the effective distance that waves would travel in parallel to the prevailing wind direction across the open water from the point of origin to the opposite shoreline in Google Maps.

### Temperature loggers

A total of four temperature loggers (HOBO Water Temp Pro v2, Onset, Cape Cod, Massachusetts, USA) were installed to measure temperature in the surface and bottom waters of the Puquios 1 and 4. Water temperature measurements were collected every 20 minutes. Two temperature loggers were installed in each lake, with the surface logger installed in the upper 5 cm of the lake. Bottom water temperature in Puquio 1 was measured at a depth of 20 cm, while temperature of the bottom waters of Puquio 4 was measured at a depth of 50 cm, consistent with average depths in both lakes [18]. Temperature logger data are presented from November 1–6, 2019 (Fig 3; S6 Fig in S1 File). In addition to presenting the results from the entire temperature dataset (*n *= 432, including surface and bottom waters in both lakes), we subsampled the logger dataset to replicate the morning and afternoon sampling periods in the discrete sample collection and in situ measurements described below. In these subsets, temperature measurements collected between 7 and 10 am (*n* = 60) were collated to represent morning sampling periods, while temperature measurements collected between 1 and 4 pm (*n *= 60) were collated to represent afternoon sampling periods matching the time frame of discrete sample collection (Table 1). Although the logger data collection period did not overlap with our campaign, analysis of weather station data suggests that air temperatures, wind speed, and gust speeds were similar between the two periods (S2 Fig in S1 File). Initial statistical evaluation indicated that the data were not normally distributed, and thus, the Mann Whitney U test was used to compare differences in temperature between the surface and bottom waters of each lake.

**Table 1:** General trends in stratification of brine chemistry of Puquio 1 and 4, November 2019. Shown here are *p*-values from Mann Whitney U-tests assessing stratification of measured parameters (Temperature, Electrical Conductivity (EC), δ^18^O and δ^2^H values). Bold font with asterisks indicates statistical significance in the evaluation of water column stratification (*p* < 0.05).

	Puquio 1 Morning	Puquio 1 Afternoon	Puquio 4 Morning	Puquio 4 Afternoon
Temperature (logger)	0.15	0.19	0.01*	0.02*
Electrical Conductivity	0.31	0.34	0.28	0.01*
δ^2^H values	0.27	0.71	0.01*	0.03*
δ^18^O values	0.15	0.89	<0.001*	0.08
δ^34^S values	0.57	0.92	0.72	0.76
Alkalinity	0.41	0.66	0.04*	0.09
Temperature (multimeter)	0.82	0.47	0.21	0.04*
pH	0.14	0.61	0.04*	0.02*
D.O.	0.13	0.11	0.01*	0.03*
Turbidity	0.79	0.47	0.60	0.01*

**Fig 2:**
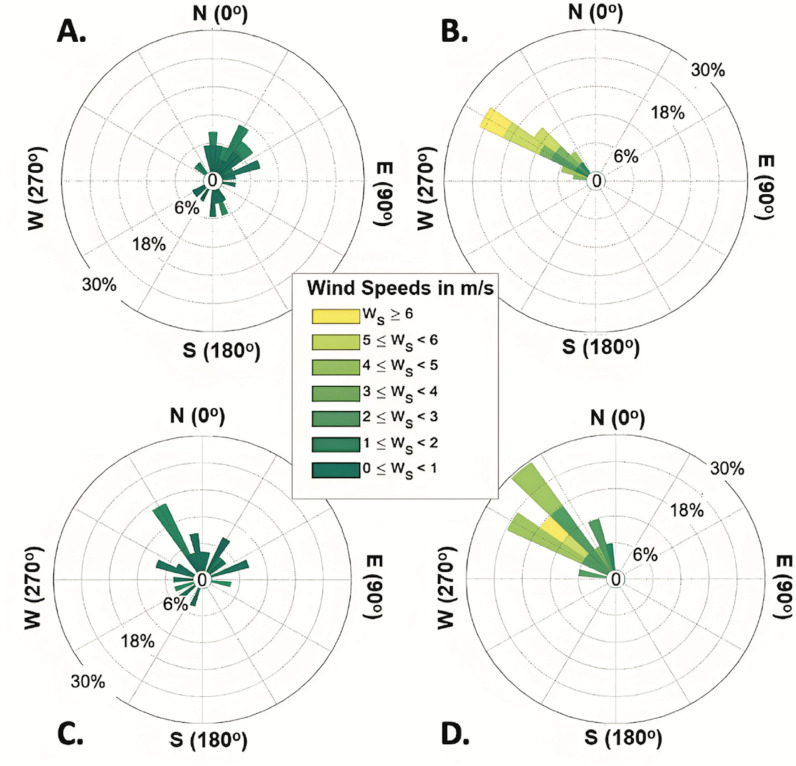
Local wind speed and environmental conditions measured using loggers installed on the weather micro station. Wind roses showing wind speed and wind direction the Salar de Llamara on November 12 (A, B) and November 13 (C, D). Morning conditions are shown in A and C, afternoon conditions are shown in B and D. Wind roses showing afternoon conditions from both days indicate an increase in wind speed as well as a more consistent (NW) direction.

### Sample collection and in situ measurements

*In situ* measurements of electrical conductivity (accuracy: ± 1 µ S/cm), pH (accuracy:0.02 pH), dissolved oxygen (D.O., accuracy: ± 0.10 ppm (mg/L)), turbidity (accuracy: ± 0.3 FNU) and temperature (accuracy: ± 0.15 °C) were collected between November 12^th^ and 13^th^, 2019 using a multimeter (HI 9829, Hanna Instruments, Rhode Island, USA) in both the surface and bottom waters of the brine pools at 26 locations (S1 Fig in S1 File) during morning and afternoon campaigns, totaling 87 *in situ* measurements for this experiment. Surface measurements were collected first to avoid disturbing stratification, and bottom measurements were collected after allowing the multimeter reading to stabilize after moving the probe to the bottom of the water column. Puquio 1 and Puquio 4 were measured at the same locations twice within a single day (‘Morning’ and ‘Afternoon’) to evaluate the change in vertical stratification of *in situ* parameters in response to changing meteorological conditions. At each measurement location, water samples were collected in 15 mL polypropylene centrifuge tubes for subsequent laboratory analysis of δ^2^H and δ^18^O values, resulting in a total of 86 measurements. *In situ* field measurements and water sampling for Puquio 1 and Puquio 4 were conducted on November 12^th^ and November 13^th^, 2019, respectively. Like the *in situ* measurements, discrete samples of brines from Puquios 1 and 4 were sampled from the same locations twice in the same day—once in morning, and again in the afternoon when the wind speed increased. Very shallow brine depths (< 0.15 m) in certain portions of Puquio 1 prevented the collection of both a surface and bottom brine sample. In these cases, only a surface brine sample was collected.

### Analysis of Oxygen and Hydrogen Isotope Values

The δ^2^H and δ^18^O values of 127 samples were analyzed on a Picarro Cavity Ring Down Spectrometer (CRDS) in the Stable Isotope Laboratory at The Rosenstiel School at the University of Miami. Sample volumes of 0.5 cc were analyzed using an injection port as previously described [17,48,49]. Four standards were run in duplicate every 25 samples. Error on the measurements was assessed as by calculating the standard deviation of repeated analyses of a standard, which were 0.05 and 0.1 ‰, for δ^18^O and δ^2^H values respectively [48]. Results are reported in reference to V-SMOW using conventional delta notation.

### Analysis of Sulfur Isotope Composition of Dissolved Sulfate

The δ^34^S value of the dissolved sulfate in all samples from the Puquios was analyzed using the ASA and Europa CFIRMS 20–20 in the Stable Isotope Laboratory at the Rosenstiel School at the University of Miami. In the field, 5 mL of a saturated BaCl_2_ solution was added to each of the brine samples collected from the Puquios, and a spontaneous reaction of BaSO4 precipitation occurred. Upon returning to the Stable Isotope Laboratory, these precipitates were filtered out of suspension and dried in a drying oven at low temperature (~ 40 °C) until completely dry. Drying times varied by sample. Next, 1 mg of the dried sample was powdered using an agate mortar and pestle and transferred into tin capsules, and vanadium pentoxide (V_2_O_5_) was added to enhance sample combustion efficiency. Three standards were similarly packed and analyzed in triplicate within each batch to assess machine drift and error on the measurement following the methods presented by Oehlert et al [50].

### Alkalinity

Alkalinity titrations were conducted using HANNA instruments HI 84531. Two commercially available standards were used to validate our method, including a low-range standard (HANNA Instruments) with an alkalinity value of 134.2 mg/L and a high standard (HANNA Instruments) with an alkalinity value of 610 mg/L, to encompass the range of alkalinity values expected in the samples. Error was assessed via 2 repeated analyses of IAPSO samples in comparison with reported reference values, and results indicate a standard deviation of ± 10 mg/L.

### Statistical analyses

Following methods presented by Bąbel [16], stratification for paired measurements collected from the surface and bottom brines at one sampling location is calculated as follows:


$$\text{S = }{\left[\text{M}\right]}_{\text{bottom}}-{\left[\text{M}\right]}_{\text{surface}}$$ (1)

where S describes the type of stratification, and [M] is the concentration of the geochemical parameter of interest. Three types of stratification can arise: normal, inverse, and not stratified [16]. Normal stratification refers to the scenario where surface waters have lower measurement values than the bottom waters, where S < B. In contrast, inverse stratification, such that S > B, occurs when surface waters have higher measurement values than the bottom waters. Non-stratified waters produce S = B and can be described as well-mixed or exhibiting a homogeneous distribution of geochemical parameters between the surface and bottom waters [16].

Mann-Whitney U tests were conducted in Matlab to calculate the degree of the stratification between the surface and bottom brines when considered as a whole. The Mann-Whitney U test assesses statistical differences between two groups on a single, ordinal variable with no specific distribution [51,52]. As applied here, *p* values less than 0.05 are considered to represent statistically significant stratification. Alternatively, *p* values > 0.5 are interpreted to represent unstratified water masses (well mixed or homogeneous).

### Results and discussion

**Fig 3:**
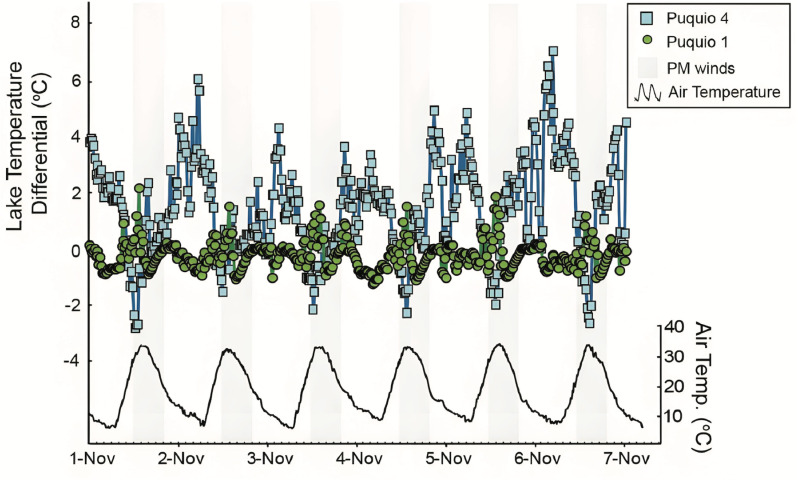
Plots of the temperature differential between surface and bottom waters of Puquio 1 (green circles) and Puquio 4 (blue squares) during the experimental period. Temperature differential was calculated by subtracting values of surface temperature from bottom temperatures measured by *in situ* loggers. Air temperature is shown in black, and grey shading highlight periods where wind speeds increased above 5 m/s as measured by the air temperature logger installed on the weather micro station.

**Fig 4:**
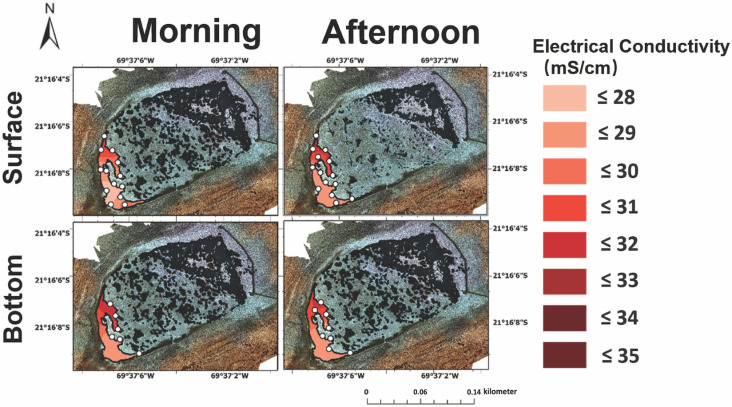
Electrical conductivity in the surface (top) and bottom (bottom) brines from Puquio 1 in both the morning (‘AM’, left) and afternoon (‘PM’, right). Measurements were collected on November 12^th^, 2019.

**Fig 5:**
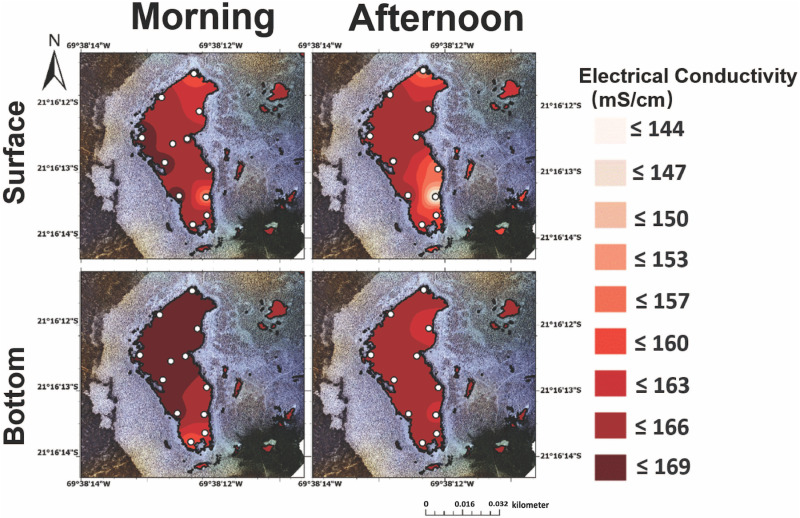
Electrical conductivity in the surface (top) and bottom (bottom) brines from Puquio 4 in both the morning (‘AM’, left) and afternoon (‘PM’, right). Measurements were collected on November 13^th^, 2019.

**Fig 6:**
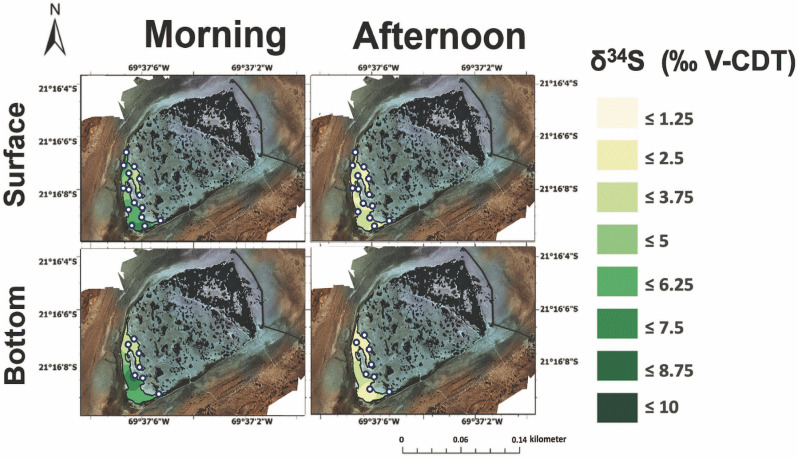
The **δ**^**34**^**S values in the surface (top) and bottom (bottom) brines from Puquio 1 in both the morning (left) and afternoon (right).** Measurements were collected on November 12^th^, 2019.

**Fig 7:**
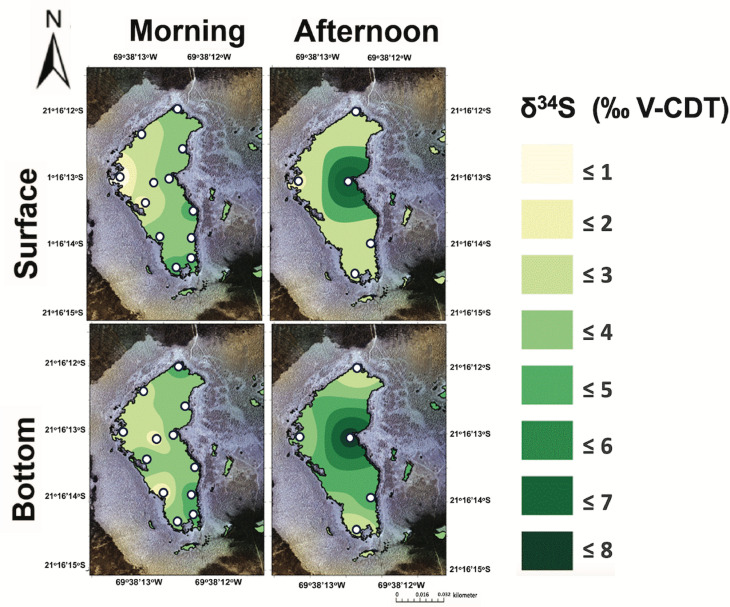
The **δ**^**34**^**S values in the surface (top) and bottom (bottom) brines from Puquio 4 in both the morning (left) and afternoon (right).** Measurements were collected on November 13^th^, 2019.

Diurnal trends in the composition and stratification in the Puquios of the Salar de Llamara varied with lake morphology; the relatively deeper steep-sided lake (Puquio 4) remained stratified in the afternoon despite an order of magnitude increase in wind speed in the afternoons (Fig 2), while measurements conducted in Puquio 1, the shallow and gently sloping lake, indicate no significant stratification of these parameters (Table 1). In the following, interplay between environmental conditions and lake morphology will be evaluated as a control on the observed trends in stratification of logger-based measurements of temperature (Fig 3) and discrete measurements of δ^34^S values of the dissolved sulfate, alkalinity, temperature, pH, DO, turbidity (Table 1), and EC (Figs 4 and 5), as well as δ^2^H, and δ^18^O values. Since δ^2^H and δ^18^O values showed similar trends (Figs 6 and 7, S3 and S4 Figs in S1 File), only δ^2^H values (Figs 6 and 7) will be presented for brevity.

### Diurnal trends in environmental conditions

Meteorological data collected by the weather micro station recorded strong diurnal cycles in environmental conditions, including wind direction, wind speed, air temperature, and relative humidity (RH) in the Salar de Llamara (S5–S7 Figs in S1 File). Within the seven days encompassing our field campaign, relative humidity ranged from less than 15 to greater than 90%, and air temperature varied between 5 and 35 °C, with diurnal cycles driving the majority of the variation magnitude (S6 Fig in S1 File). Wind speeds ranged from < 1 to 9.9 m/s, and while gust speeds were variable in intensity throughout the day, afternoon gust speeds reached velocities > 13 m/s (Fig 2, S6 Fig in S1 File). Observations of higher wind speed and gustiness in the Salar de Llamara during the afternoon are consistent with previous results of diurnal variability in wind speed conducted in the hyperarid core of the Atacama Desert [53] and the Salar de Huasco [54].

Measurements of environmental conditions collected in the morning and afternoon (Fig 2, S6 and S8 Figs in S1 File) suggest the occurrence of two environmental regimes: relatively calm mornings, and windy or gusty afternoons. Observations from our field campaign in November 2019 support this regime delineation; lake waters in the Puquios were relatively calm in the morning when average wind velocity was 0.65 m/s (Fig 2, S6 Fig in S1 File), while visible disturbance of the water column was observed when wind speeds increased in the afternoon by an order of magnitude to an average velocity of 6.94 m/s (Fig 2, S6 Fig in S1 File). *In situ* measurements showed that afternoon wind direction reaches a steady state (variability < 45 ø, average direction North-West 315°), a change which accompanies an increase in wind speed and increased air temperatures (Figs 2 and 3). Thus, mornings, characterized by relatively low wind speeds, rising temperatures, and decreasing RH, contrast with high wind speeds, peak air temperature, and low RH in the afternoon (S6 Fig in S1 File).

### Diurnal trends in lake chemistry

Measurements of temperature collected by the HOBO logger from the surface waters of Puquio 1 ranged from 15.1 to 30.95 °C (Fig 3), similar to those recorded in the surface waters of Puquio 4 (14.75 to 30.82 °C, Fig 3) during the logging period (green box, S6 Fig in S1 File). Bottom waters exhibited similar maximum temperatures for Puquio 1 (30.4 °C) and Puquio 4 (30.8 °C), but the minimum temperatures recorded in the bottom waters of Puquio 4 were ~ 5 degrees warmer than those in Puquio 1 (Fig 3, S6, S7 Figs, and S1 Table in S1 File). Colder air temperatures have primarily cooled the surface waters of Puquio 4, with minimal impact on the bottom waters (S4 Fig in S1 File). In contrast, both surface and bottom measurements of temperature chill to approximately the same degree in Puquio 1 overnight, thus the diurnal temperature variation in the shallow water column of Puquio 1 exhibits a larger range in temperature than Puquio 4 which is characterized by deeper water depths [18]. Compared to the median historical temperature measurements collected in Puquio 1 in October 2011, March 2012, November 2017, and January 2018 [12,17,18], diurnal temperature ranges in Puquio 1 represent ~ 15.6 % of annual temperature variability. Limited publication of temperature measurements in Puquio 4 precludes similar analysis. The minimum, maximum, average and medium values of lake waters in Puquio 1 and 4 from November 1^st^ to November 6^th^ are presented in the S1 table in S1 File.

In terms of electrical conductivity, surface and bottom waters in Puquio 1 exhibited a range between 26.7 to 31.4 mS/cm and 27.5 mS/cm to 31.6 mS/cm, respectively, in EC from south to north. The highest EC values in Puquio 1 (Fig 4, 31.6 mS/cm) were measured in both the morning and afternoon and occurred in the northern part of the lake. As expected, EC values in Puquio 4 were significantly higher than those in Puquio 1 (Figs 4 and 5), an observation consistent with previous measurements in the Salar de Llamara [17,18]. Surface and bottom measurements of saline brines from Puquio 4 ranged from 152.7 mS/cm to 167.1 mS/cm and 156.5 mS/cm to 166.7 mS/cm, respectively (Fig 5). The highest EC values in Puquio 4 were consistently observed on the western margin of the saline lake, while the lowest EC values were observed in the surface waters on the eastern margin of the lake during the afternoon (Fig 5). Notably, although temperature stratification in Puquio 4 was reduced during the afternoon sampling periods (S5–S7 Figs in S1 File), stratification in electrical conductivity was promoted in Puquio 4, indicating that temperature and salinity stratification can be decoupled on diurnal time frames. However, temperature can exert an important control on density, stratification [19,55], evaporation rates [*i.e.,* 53], and thus the chemical composition and salinity of the brine. In concert with temperature, chemical characteristics such as elemental concentrations, total dissolved solids, and conductivity impact mineral deposition through thermodynamic controls on solubility, such that annual cycles of mineral precipitation and dissolution may result from changes in temperature of the water masses [56]. For instance, gypsum solubility was found to increase significantly with decreasing temperature in a recent laboratory-based study conducted between 0–40 °C [57]. Depending on the locus of mineral formation, whether in the surficial waters, the chemocline, or at the sediment-water interface, temperature control on mineral solubility can induce changes in sedimentological and petrographic archives that provide records of changing environmental conditions through time [15,56]. Compared to the median historical EC measurements collected in Puquio 1 in October 2011, March 2012, November 2017, and January 2018 [12,17,18], diurnal EC ranges in Puquio 1 represent ~ 23.9% of annual EC variability, while EC variability measured during this field campaign represents ~ 2.9% of annual ranges.

The δ^34^S values of the dissolved sulfate exhibited spatial heterogeneity across Puquios 1 and 4. In brines from Puquio 1, the δ^34^S values of the dissolved sulfate (Fig 6, averages in S2 Table in S1 File) were typically positive, and ranged from + 1 to + 9 ‰ V-CDT, with some measurements higher than those previously reported for a transect of samples spanning Puquio 1, the Transition Zone and Puquio 2 [12]. No prior measurements of δ^34^S values of dissolved sulfate have been reported for Puquio 4, but our new values ranged from + 1 to greater than + 7 δ^34^S ‰ V-CDT depending on the water depth and time of day (Fig 7, averages in S3 Table in S1 File). The ranges in δ^34^S values of the dissolved sulfate from our study are consistent with prior measurements of δ^34^S values of dissolved sulfate, soil sulfates, and sulfate minerals from the region [12,58–61]. Atmospheric deposition (+ 2.5 ‰ V-CDT, [62]), weathering products of Andean rocks transported streams, lakes and evaporite salts forming in salar environments (+ 2.9 to + 8.7 ‰ V-CDT, [58,59,61]), underlying evaporites like anhydrite in the Soledad formation (+ 6 ‰ V-CDT; [60,63]), and marine sulfate (+ 21 ‰ V-CDT; [64–66]) have all been cited as part of a mixture of sources that supply sulfur to salar environments in the Atacama Desert ([58–61,67]). In contrast, a recent multi-isotope analysis suggest bulk marine aerosol deposition can conceivably be the sole source of the majority of sulfate in the region [68].

Diurnal variability in average δ^34^S values of dissolved sulfate in Puquio 1 (S2 Tables in S1 File) suggests that processes other than changes in sulfur source could also be important. Localized gypsum precipitation and the metabolic activity of microbial communities in these settings could be possible explanations. Bottom types in Puquio 1 are microbial in origin, forming loose and flocculent sediment comprised of small irregular gypsum precipitates, carbonate, magnesium clays, with an abundance of microbes and extracellular polymeric substances (EPS) [17,18,23,37]. The ~ 2.7 ‰ shift towards lower δ^34^S values of the dissolved sulfate in Puquio 1 from morning to afternoon (S2 Table in S1 File) is a significant diurnal change (Mann Whitney U test, *p* value = 0.0014 for Puquio 1 surface waters), but precipitation of gypsum cannot fully explain this shift since the mineralogical fractionation factor (α_gypsum-brine_) is only 1.00165, with measured Δ^34^S_precipitate-brine_ between + 1.59 and + 1.65 ‰ V-CDT [69,70].

Microbial sulfur cycling may supplement or potentially explain the entirety of diurnal changes in δ^34^S values of the brines from morning to afternoon in Puquio 1. Microbial communities are not homogeneously distributed throughout an environment and microbial activity and metabolic pathways are known to change along environmental gradients [71–75], and Puquio 1 experiences more substantial changes in temperature and electrical conductivity than Puquio 4 (Figs 3–5). Free-living microbial communities inhabiting the brines in Puquio 1 employ sulfur cycling metabolisms, including sulfide oxidation and sulfate reduction, (*i.e.,* [18]), which can induce sulfur isotope fractionation ([76,77], among others). For example, microbial sulfide oxidizers are known to generate $\Delta_{34}S_{SO_{4}^{2-}-H_{2}S}$ between - 5 and + 5 ‰ V-CDT ([78] and references therein). Bacterial sulfate reduction is commonly observed to occur in hypersaline terrestrial systems [79–84], including the Puquios [18], and can affect the concentration of dissolved sulfate [77,85–87] as well as the δ^34^S values [76,77,88,89]. This is consistent with our observations since the highest δ^34^S values were observed in Puquio 1, which dominantly consists of a microbial bottom type [12,17,18,23]. Microbial sulfate reduction is also known to impact carbonate mineral precipitation [90], a possibility that is consistent with the mineralogy of precipitates forming in Puquio 1, as well as the diurnal changes in pH, dissolved oxygen, and alkalinity observed in this system (Table 1, S2 Table in S1 File).

Changes in average δ^34^S values of the dissolved sulfate in Puquio 4 (0.3–0.6 ‰, depending on depth, Tables 1, S3 Table in S1 File) were statistically insignificant (Mann-Whitney U test, *p* > 0.05), and may be a product of the larger reservoir of dissolved sulfate in Puquio 4 compared to Puquio 1 [17]. Thus, rates of mineral deposition or metabolic activity of the microbial communities may be insufficient to fractionate the δ^34^S values of the larger pool of dissolved sulfate in Puquio 4, an explanation that is supported by substantially reduced evidence of microbial cells and EPS noted in prior studies [17,18] and the fact that sulfate reduction was not a predicted metabolism for free-living microbial communities in Puquio 4 [18]. Future investigations employing a multi-proxy geochemical approach (*i.e.,* [68]) coupled with time series measurements of microbial community activity (*i.e.,* [91–93]) would be useful to pursue this hypothesis.

**Fig 8:**
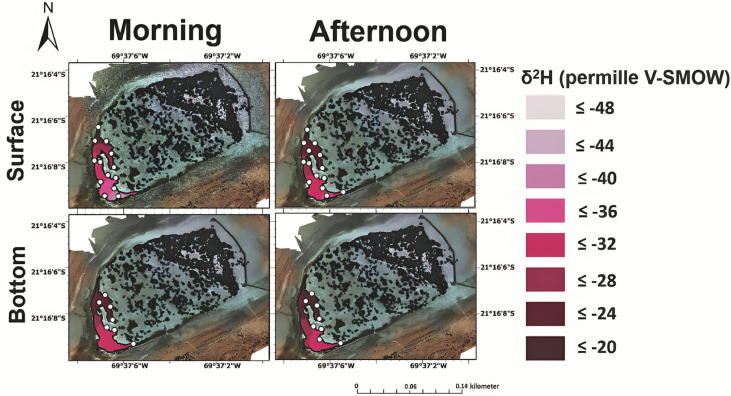
δ^2^H values in the surface (top) and bottom (bottom) brines from Puquio 1 in both the morning (left) and afternoon (right). Measurements were collected on November 12th, 2019.

**Fig 9:**
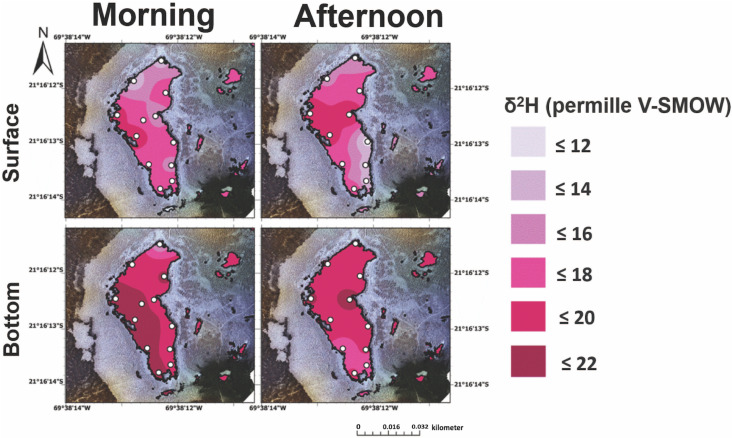
δ^2^H values in the surface (top) and bottom (bottom) brines from Puquio 4 in both the morning (left) and afternoon (right). Measurements were collected on November 13th, 2019.

Measurement of δ^2^H values indicates a high degree of spatial heterogeneity in both lakes, with lateral, north-to-south gradients observed in Puquios 1 and 4 (Figs 8 and 9). Both lakes also exhibit significantly higher values for δ^2^H and δ^18^O than published ranges for rainfall indicated by the local meteoric water line (LMWL) proposed for this region (Fig 8) [94–96] consistent with previous samples collected during different seasons [12,17,18]. Evaporation thus plays an important role in producing the δ^2^H and δ^18^O values of waters in Puquio 1 and 4. Regression of δ^2^H and δ^18^O values was used to calculate the intercepts with the Local Meteoric Water Line (LMWL) providing an indication of the stable isotopic composition of the original source water (S9 Fig in S1 File). A similar source water is indicated for Puquio 1 and Puquio 4 with the exception of surface waters collected in the morning (S9A Fig in S1 File), confirming previous interpretations of strong evaporative control [94,96] on this system prior to emerging in the Puquios.

### Diurnal trends in lake stratification

Trends in water column stratification in the saline lakes of the Salar de Llamara varied through space and time. Significant stratification in EC, alkalinity, temperature, pH, dissolved oxygen, δ^2^H and δ^18^O values, and turbidity, were only observed in Puquio 4, and no significant stratification of these parameters was observed in Puquio 1 (Table 1), providing insight into the dynamics of lake stratification with respect to lake morphology. Two-tailed t-tests revealed that temperature in Puquio 4 was stratified (*n* = 432, *p* < 0.01), while those in Puquio 1 were not (*n* = 432, *p* > 0.05, Table 1), with waters in Puquio 4 particularly stratified in the late afternoon and especially overnight. Temperature stratification in saline lakes is an important control on hydrology, density stratification, mineral deposition, nutrient availability, and biological function. Relevant to the temperature tolerance of resident free-living and benthic communities in saline lakes, thermal stratification can also impact the exchange of oxygen and essential nutrients, thus influencing ecosystem structure, function, and nutrient mineralization [*i.e*., 79] The rapid thermal equilibration of surface and bottom waters in Puquio 1 may thus facilitate nutrient supply that accommodates the formation of microbial substrates [12,17,18,23,97,98] in this shallow, gently sloping, and low salinity lake. In addition to nutrient and oxygen availability, other impacts on ecosystem structure, including species diversity and the development of biological niches, can be induced by temperature variations and thermal stratification.

### Impacts of environmental conditions and lake morphology on stratification

Changes in wind direction, speed, and air temperature are important drivers of saline lake stratification, evaporation, and vertical mixing, making lakes sensitive to changes in environmental conditions through time [16,99,100]. Like previous studies [12,17,34], new data presented here shows that evaporation plays an important role in determining the chemical composition of the Puquios. Indeed, estimates of evaporative loss fraction ranged from 0.71 to 0.87 [17] when δ^2^H and δ^18^O values measured on saline lake brines were used to parameterize a statistical model assessing the degree of evaporation (Hydrocalculator 1.03; [101]). Since evaporation can create differences in chemistry and stratification, it can also influence the style of sediment deposition. Criado-Reyes et al. [21] showed how the morphogenetic pathways of gypsum structures in Puquio 2 were influenced by density stratification driving changes in brine chemistry, particularly undersaturated zones that facilitated dissolution of gypsum at the base of these structures.

Previous studies have attributed impacts of wind velocity and constancy of wind direction to the development of stratification in lakes [44,102,99]. Large shallow lakes are known to be impacted by wind-wave disturbance [103–105], which can affect resuspended sediment loads [104,106] and nutrient availability [107]. Wind-waves can also induce periods of mixis and overturning in shallow polymictic lakes [16,108]. In the Salar de Llamara, shallow, gently sloping lakes like Puquio 1 were proposed to be well mixed and characterized by relatively low EC values [17,18]. Temperature and EC variability within a single day in Puquio 1 accounts for 15.6 % to 23.9 % of the annual ranges observed. While diurnal temperature changes are common in all lakes, the fluctuations noted here are pronounced in shallow and gently sloping lakes like those found in modern saline lakes like the Great Salt Lake [109,110] ancient saline lake settings [111–113]. Modern low-slope gradient lakes tend to correlate with microbial substrates, both in the Puquios [12,17,18,23], and also other shallow, gently sloping saline lake environments around the world [6,114–117]. Previous workers have attributed a similar lack of lake stratification in saline lake environments hosting microbial communities to wind-driven mixing [114]. In a systematic investigation of more than 60 shallow lakes in Western Australia, neither temperature nor salinity stratification was observed in shallow lakes [118], supporting the interpretation proposed here.

Lateral physico-chemical gradients have also been shown to influence the activity and composition of the free-living biota and microbial community structure, and thus can influence sedimentation style, mineralogy, and likely the preservation potential of such depositional systems through time [17,23]. Importantly, the spatial variability observed in the geochemical characterization conducted here within a single week approximates the range in such parameters induced by seasonal changes in climate, wind, and environmental conditions through longer observation periods [17,119]. These results demonstrate the dynamic and heterogeneous nature of polyextreme salar environments, the culmination of which is known to lead to specific sedimentation patterns in evaporite environments [16–18,115,120–122].

Relatively deeper and steeper sided lakes in the Salar de Llamara like Puquios 2 and 4 were proposed to facilitate stratification [18], one characteristic that has been attributed to the deposition of crystalline bottom types dominated by bladed selenite crystals [16,23,24]. Similar crystalline bottom types were reported in other lake systems outside of the Puquios, including in the Salars de Gorbea and Ignorado [115], the Salar de Carcote [2], Barros Negros [2,123], Pozo Bravo [124], Laguna Verde [2], and Longar Lake in Spain [117] in salterns such as the EMISAL salt works located in Egypt [125], the Dhahban solar saltwork [126] and Guerrero Negro located on Mexico’s Baja California Peninsula [127,128]. Although one study indicated a lack of water column stratification [115], others have proposed that water column stratification plays an important role in shaping crystal morphology and mineralogy [16,121], sculpting gypsum structures [21], and producing environments favorable to microbialite development [129]. New insights produced here suggest that heterogeneity in water column chemistry, including stratification, can occur over short time scales, providing motivation for further study of lake water stratification as a constructive and/or destructive force in mineral deposition and preservation over time.

### Implications for the geological record

The influence of diurnal changes in wind speed and direction on the stratification of saline lakes in the Salar de Llamara holds implications for studies of extreme environments and microbial communities more broadly. The results of this study improve understanding of the relationship between lake morphology and environmental conditions, including diurnal variations in wind speed and temperature, and assess their impact on saline lake stratification [12,17,18]. Measurements conducted here showed that Puquio 4, a relatively deep and steep sided lake in this system, maintained stratified water masses with respect to temperature, alkalinity, pH, dissolved oxygen, EC, δ^2^H, and δ^18^O values on diurnal timescales, despite an order of magnitude increase in wind velocity occurring in the afternoon (Fig 2, S9 Fig, S3 Table in S1 File). Increased wind speed produced a well-mixed water column in the shallow, gently sloping Puquio 1 (S2 Table in S1 File) with environmental conditions varying on a diurnal scale that are 15.6 – 23.9% of annual variability. Since stratification has previously been linked to styles of mineral deposition and the relationship of minerals with benthic microbial communities in the saline lakes of the Salar de Llamara [17,18,21] and other saline lakes globally [16,121,130], resistance to diurnal perturbations in environmental conditions may be one important characteristic of depositional settings that facilitate evaporite mineral deposition with minor microbial influence. Our observations linking lake morphology, stratification, and the style of evaporite mineral deposition are relevant to the definition of facies models for evaporitic environments, the interpretation of biogeochemical records derived from evaporite minerals, and the ongoing assessment of controls on biosignature preservation in saline lake environments on Earth [16,49,121,130–133]. Improved understanding of the dynamics of these terrestrial systems can provide better analogues for interpreting conditions persisting at the edge of habitability [2, and references therein], as well as conditions on early Mars that may have generated paleolake evaporite-carbonate deposits in the Jezero Crater [63,133–135].

## Conclusions

Here, we documented dynamic changes in temperature, electrical conductivity, pH, dissolved oxygen, alkalinity, and stable hydrogen, oxygen, and sulfur isotope values occurring daily in saline lakes found in an extreme environment from the Atacama Desert. These lake environments are important depositional analogues for evaporite mineral depositional systems in the geological record of Earth, as well as in extraterrestrial settings. Results indicate that environmental conditions, such as wind speed and wind direction, interact with lake morphology to facilitate water column stratification or mixing. Despite order-of-magnitude increases in afternoon wind speed, the relatively deeper and steep-sided lake maintained stratification during the experiment. In contrast, the waters in the shallow, gently sloping lake were well-mixed by the change in wind speed and exhibited diurnal variations equating to ~ 20% of the seasonal ranges published previously. Furthermore, spatial trends in water column stratification, including in electrical conductivity, stable oxygen, hydrogen, and sulfur isotope values, varied over time periods less than a day within a single salar environment. Such lateral gradients in brine chemistry are known to play an essential role in determining microbial activity, nutrient dynamics, and mineral-water interaction, all factors impacting the deposition and morphology of minerals in salar environments around the world. From these results, we infer that microbial ecosystems inhabiting saline lake settings in the salars of South America likely tolerate wider ranges in environmental conditions than previously appreciated, and that evaporite sedimentation represents the culmination of depositional processes occurring across multiple time scales.

## Supporting information

S1 File The supplementary material for this article includes three tables (S1 -S3 Tables) and nine figures (S1-S9 Fig.). (DOCX)
