# Supplementary material for: Lake morphology and meteorological conditions impact stratification of saline lakes in the Atacama Desert
Source: PLoS One. 2025 May 5;20(5):e0321759. doi: 10.1371/journal.pone.0321759 (PMC12052196; doi:10.1371/journal.pone.0321759)
Supplement: S1 File — (DOCX) [file pone.0321759.s001.docx]

*Supporting information for:*

**Lake morphology and meteorological conditions impact stratification of saline lakes in the Atacama Desert**

Tianshu Kong^1^, R. Pamela Reid^1^, Erica P. Suosaari^2^, Daniela Maizel^3^, Luis. R. Daza^4^, Alvaro T. Palma^4^, Amanda M. Oehlert^1^*

^1^Department of Marine Geosciences, Rosenstiel School of Marine, Atmospheric, and Earth Science, University of Miami, Miami, FL 33149, U.S.A

^2^Department of Mineral Sciences, National Museum of Natural History, Smithsonian Institution, Washington, District of Columbia 20002, U.S.A

^3^Department of Ocean Sciences, Rosenstiel School of Marine, Atmospheric, and Earth Science, University of Miami, Miami, FL 33149, U.S.A

^4^Fisioaqua, Las Condes, 6513677 Santiago, Chile

*Corresponding author:

Email: [aoehlert@miami.edu](mailto:aoehlert@miami.edu)

**Introduction**

Supplementary material for this article includes three tables, nine figures, and three open access datasets available as .xlsx files from the Mendeley Open Access Data Repository listed below.

**Contents of this file:**

Supplementary Tables S1 - S3

Supplementary Figures S1 – S9

**Additional Supporting Information**

Open access datasets available here as a Mendeley Open Access Data Repository (Kong et al., 2024) with CC BY 4.0 license:

Kong, T.; Reid, R. P.; Suosaari, E.P.; Maizel, D., Palma, A.T.; Daza, R.; Oehlert, A.M. “Dataset: Geochemistry and Meteorological Data from the Salar de Llamara”, Mendeley Data, 2024, V1, doi: 10.17632/knhnhf8v4p.1

**SUPPLEMENTARY TABLES**

**S1 Table**. Minimum, maximum, average, and median temperature measurements collected by the loggers, as well as the calculated temperature differential for the surface and bottom brines for both Puquios 1 and 4.

| Temperature (^o^C) | Puquio 1 Surface | Puquio 1 Bottom | Temperature  Differential | Puquio 4 Surface | Puquio 4 Bottom | Temperature  Differential |
| --- | --- | --- | --- | --- | --- | --- |
| n | 432 | 432 | 432 | 432 | 432 | 432 |
| Minimum | 15.1 | 14.5 | -1.2 | 14.7 | 19.1 | -2.8 |
| Maximum | 30.9 | 30.4 | 2.00 | 30.5 | 30.8 | 6.8 |
| Average | 21.2 | 20.9 | -0.3 | 22.9 | 24.2 | 1.3 |
| Median | 19.7 | 19.3 | -0.3 | 22.6 | 23.6 | 1.3 |

**S2 Table**. Average values (± standard deviation) for the surface and bottom brines for each chemical parameter studied, shown for both the morning (AM) and afternoon (PM) for Puquio 1.

| Chemical Parameter | AM  Surface | AM  Bottom | Stratification (%Difference) | PM  Surface | PM  Bottom | Stratification (%Difference) |
| --- | --- | --- | --- | --- | --- | --- |
| n | 13 | 6 |  | 13 | 8 |  |
| EC (mS/cm) | 28.9  ± 15.6 | 29.7  ± 19.4 | Normal  (2.6) | 29.9  ± 19.3 | 29.2  ± 16.7 | Inverse  (2.2) |
| δ^2^H (‰ VSMOW) | -33.8  ± 7.4 | -30.4  ± 6.3 | Normal  (11.3) | -29.4  ± 5.8 | -28.4  ± 6.0 | Normal  (3.6) |
| δ^18^O (‰VSMOW) | -2.4  ± 1.6 | -1.6  ± 1.6 | Normal  (56.8) | -1.3  ± 1.5 | -1.2  ± 1.4 | Normal  (12.8) |
| δ^34^S (‰VCDT) | 5.2  ± 2.1 | 4.6  ± 2.8 | Inverse  (13.5) | 2.5  ± 1.3 | 2.6  ± 1.2 | Normal  (3.1) |
| Alkalinity (mg/L) | 141.4  ± 14.1 | 155.6  ± 37.7 | Normal  (9.1) | 133.7  ± 6.5 | 134.5  ± 2.6 | Normal  (0.6) |
| Temperature (°C) | 23.1  ± 2.5 | 23.6  ± 1.6 | Normal  (2.1) | 26.0  ± 2.0 | 25.2  ± 0.9 | Inverse  (3.2) |
| pH | 8.2  ± 0.08 | 8.3  ± 0.03 | Normal  (0.5) | 8.3  ± 0.1 | 8.3  ± 0.1 | Normal  (0.1) |
| D.O. (mg/L) | 5.6  ± 1.1 | 6.5  ± 0.8 | Normal  (13.7) | 5.9  ± 0.7 | 5.5  ± 0.3 | Inverse  (6.5) |
| Turbidity (FNU) | 18.2  ± 26.2 | 6.7  ± 3.6 | Inverse  (173.1) | 19.4  ± 32.9 | 15.6  ± 21.7 | Inverse  (24.9) |

**S3 Table.** Average values for the surface and bottom brines for each chemical parameter studied, shown for both the morning (AM) and afternoon (PM) for Puquio 4.

| Chemical Parameter | AM  Surface | AM  Bottom | Stratification (%Difference) | PM  Surface | PM  Bottom | Stratification (%Difference) |
| --- | --- | --- | --- | --- | --- | --- |
| n | 12 | 12 |  | 11 | 11 |  |
| EC (mS/cm) | 162.9  ± 43.3 | 164.3  ± 32.2 | Normal  (0.9) | 160.0  ± 70.4 | 165.1  ±18.9 | Normal  (3.12) |
| δ^2^H (‰ VSMOW) | 16.4  ± 2.3 | 19.7  ± 1.7 | Normal  (16.6) | 16.2  ± 3.0 | 18.9  ± 1.5 | Normal  (14.04) |
| δ^18^O (‰ VSMOW) | 9.0  ± 1.0 | 10.0  0.3 | Normal  (9.7) | 8.4  ± 2.2 | 9.5  ± 1.3 | Normal  (12.47) |
| δ^34^S (‰VCDT) | 3.1  ± 1.3 | 3.3  ± 1.3 | Normal  (4.6) | 3.4  ± 2.0 | 3.9  ± 2.1 | Normal  (12.18) |
| Alkalinity (mg/L) | 675.6  ± 28.3 | 699.6  ± 18.6 | Normal  (3.4) | 620.4  ±52.9 | 677.8  9.4 | Normal  (8.46) |
| Temperature (°C) | 22.5  ± 1.5 | 23.1  ± 2.3 | Normal  (2.9) | 28.8  ± 2.7 | 30.3  ± 2.0 | Normal  (4.96) |
| pH | 8.1  ± 0.02 | 8.0  ± 0.02 | Inverse  (0.3) | 8.0  ± 0.04 | 8.0  ± 0.02 | Inverse  (0.50) |
| D.O. (mg/L) | 1.9  ± 0.3 | 1.5  ± 0.3 | Inverse  (24.5) | 2.2  ± 0.5 | 1.8  ± 0.3 | Inverse  (22.35) |
| Turbidity (FNU) | 1.6  ± 1.2 | 5.5  ± 14.6 | Normal  (71.8) | 1.9  ± 1.9 | 2.9  ± 1.7 | Normal  (32.98) |

**SUPPLEMENTARY FIGURES**

**
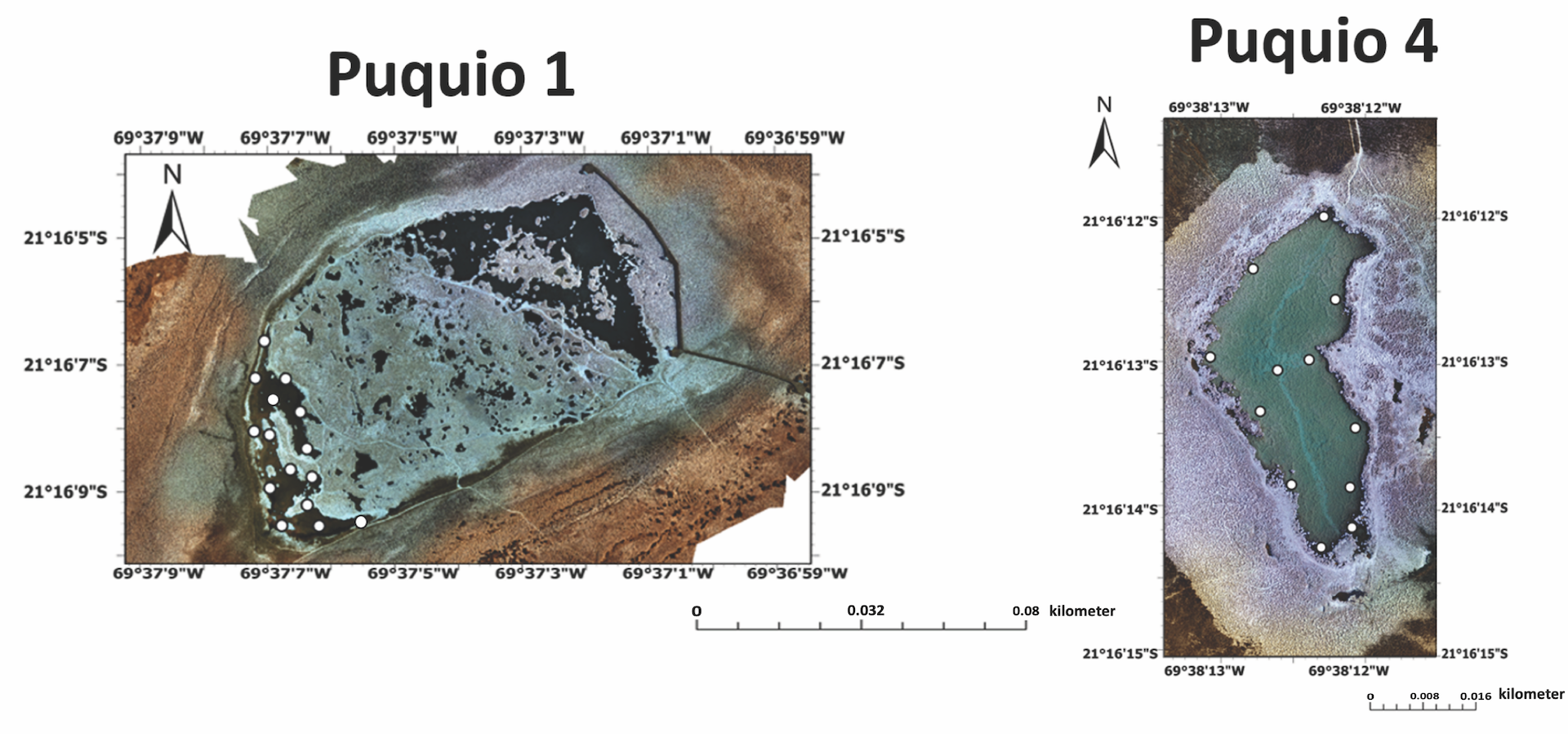
**

**S1 Fig. Sampling locations.** White circles show sample locations from Puquio 1 and Puquio 4 for the morning and afternoon measurements. Because of shallow water depths in Puquio 1, not all sampling stations were deep enough for surface and bottom measurements.

**
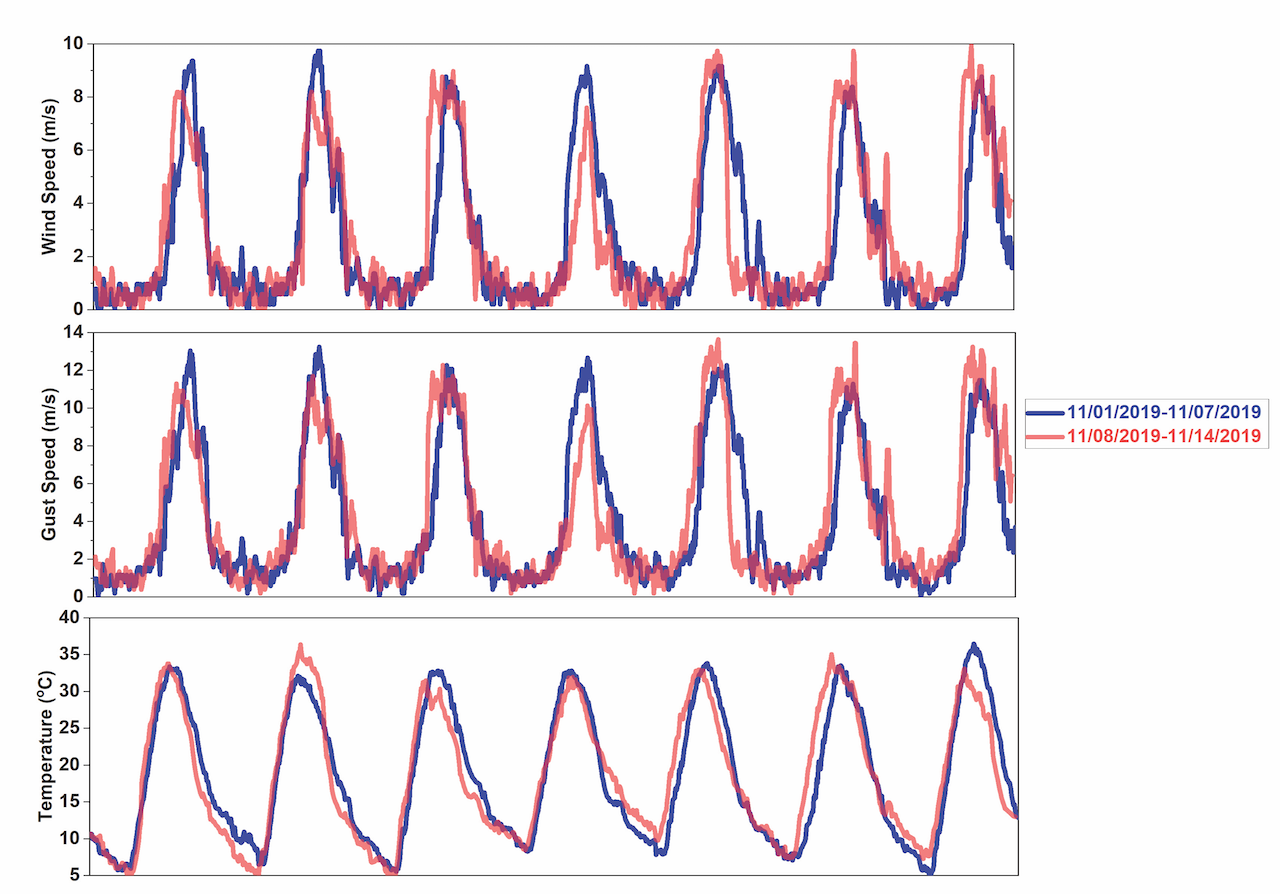
**

**S2 Fig. Comparison of weekly trends in meteorological data.** Plots of diurnal variability in wind speed, gust speed, and air temperature between the week November 1–7, 2019 and the week November 8–14, 2019.


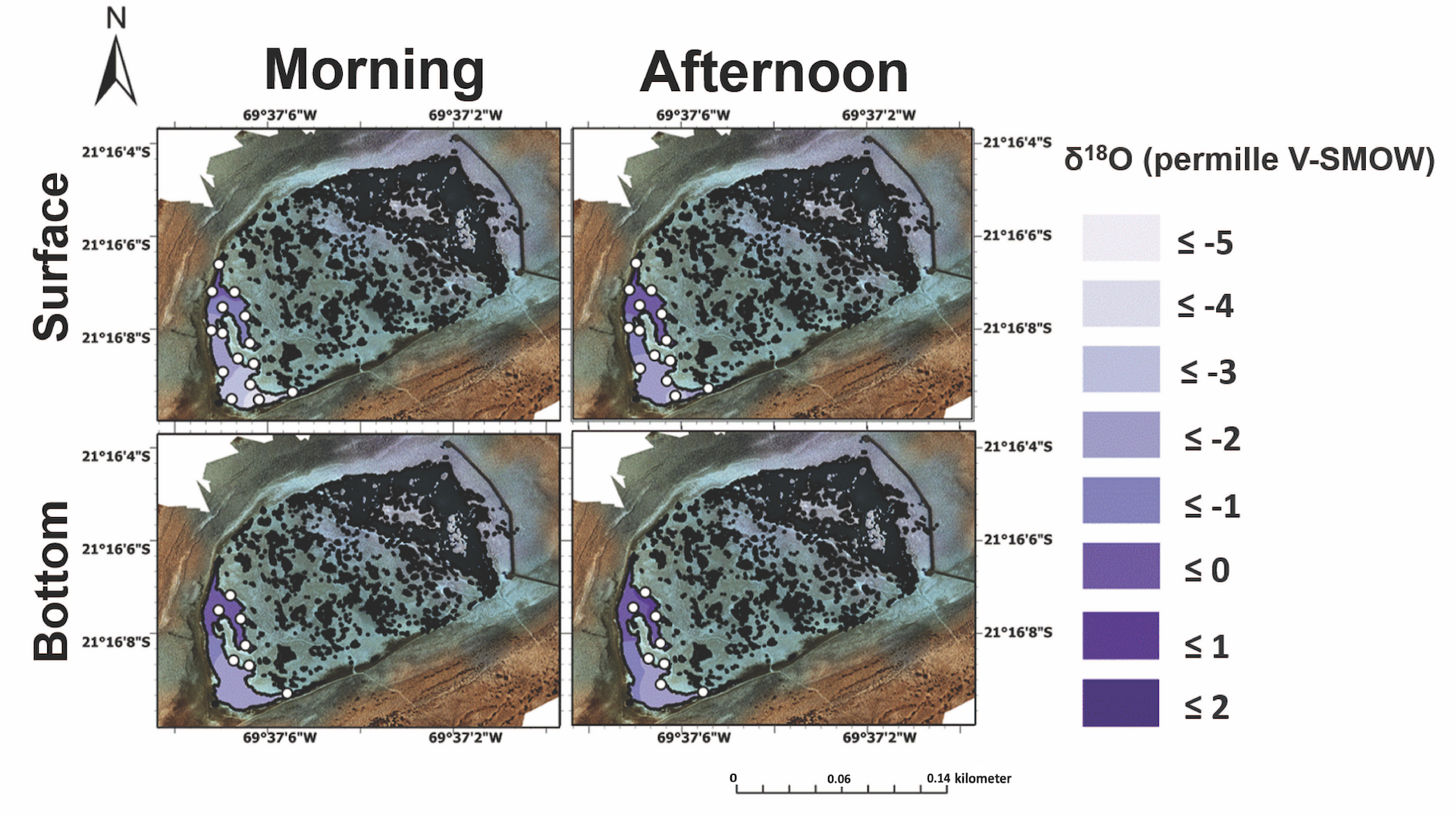


**S3 Fig. δ^18^O values ranges in the surface (top) and bottom (bottom) brines from Puquio 1 in both the morning (left) and afternoon (right).** Measurements were collected on November 12^th^, 2019.


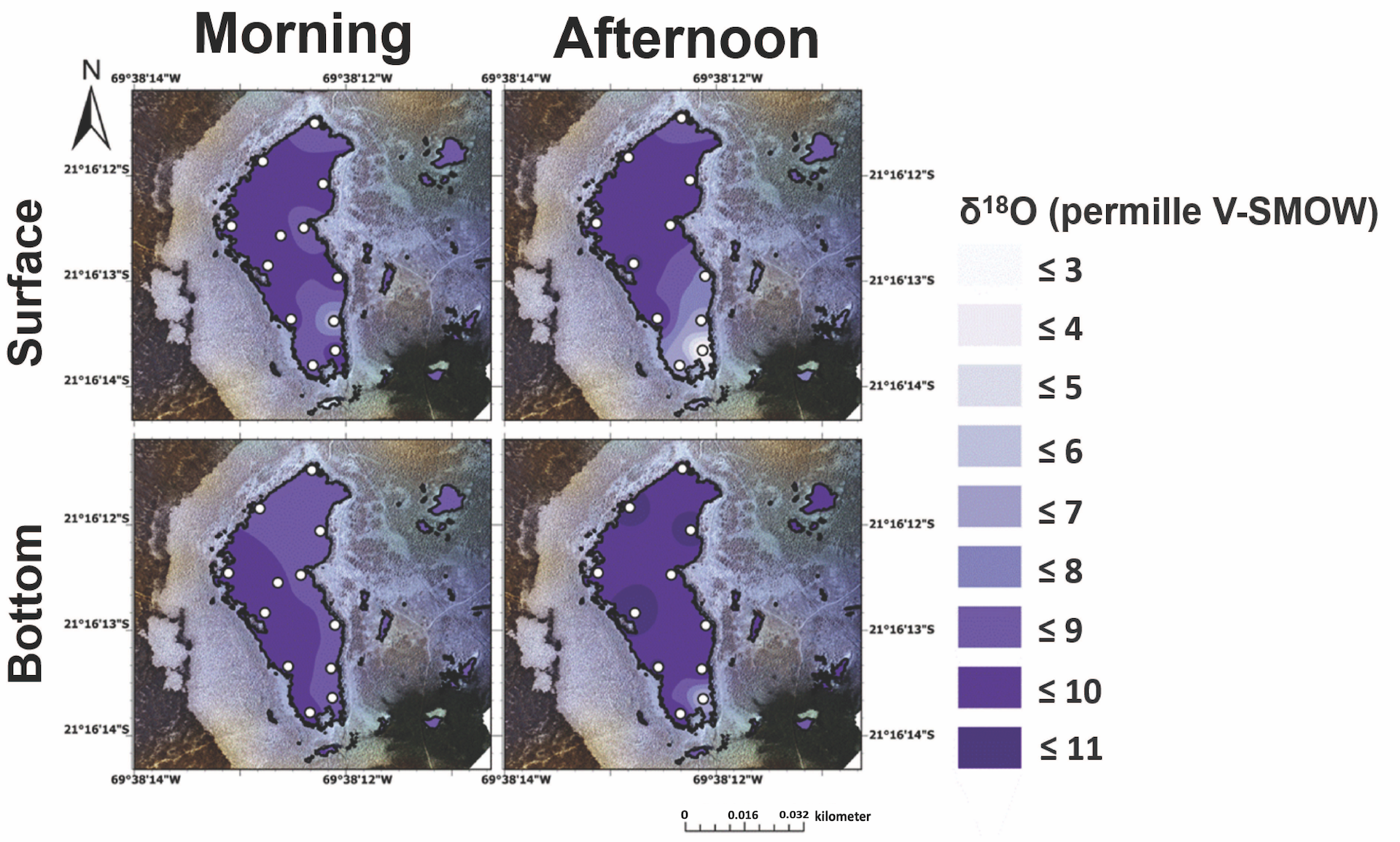


**S4 Fig. δ^18^O values range in the surface (top) and bottom (bottom) brines from Puquio 4 in both the morning (left) and afternoon (right).** Measurements were collected on November 13^th^, 2019.


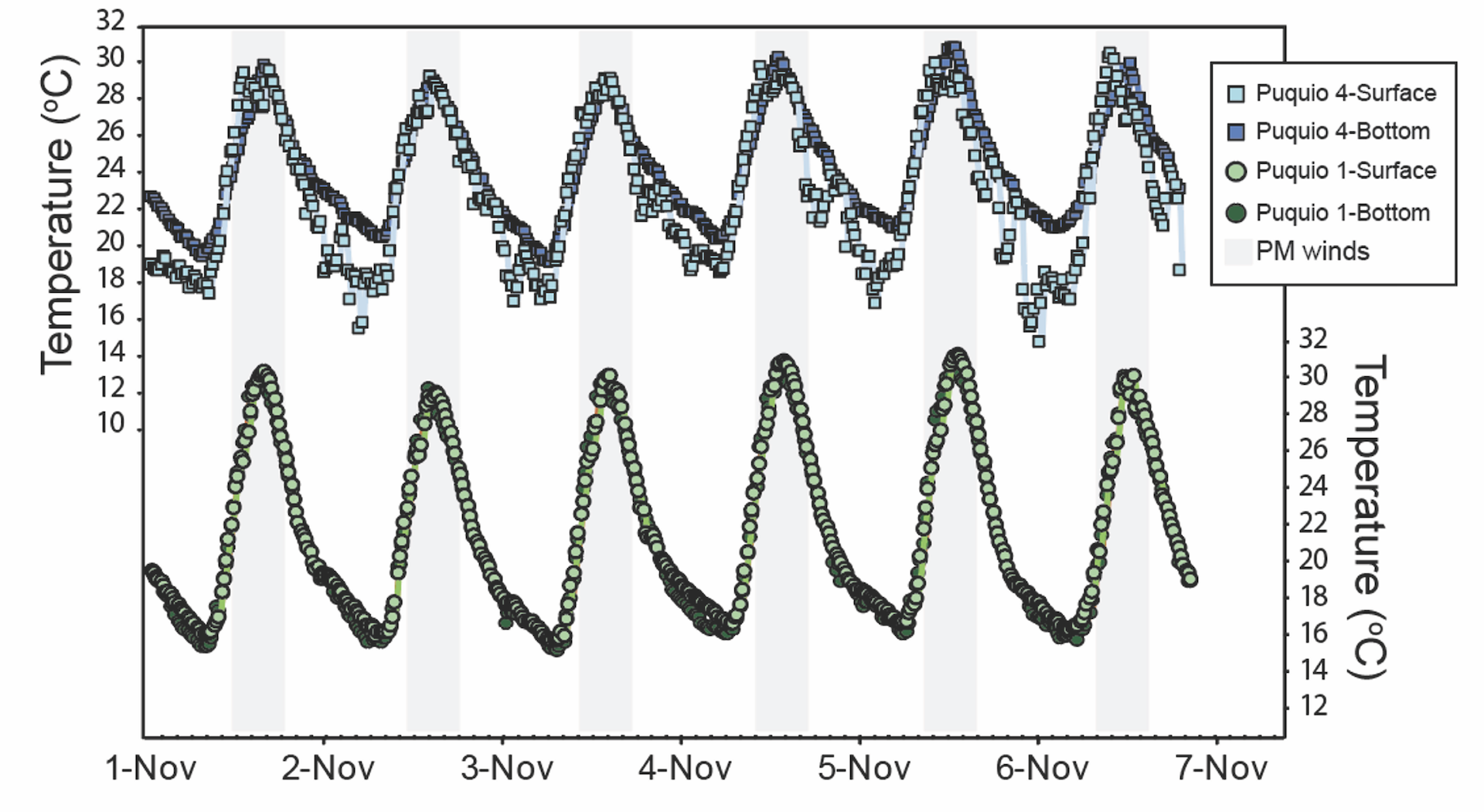


**S5 Fig. Temperature measurements of surface (light blue squares) and bottom (dark blue squares) waters from Puquio 4 are shown on the top of the figure, significant episodes of thermal stratification are observed overnight.** Temperature measurements of surface (light green circles) and bottom (dark green circles) waters from Puquio 1 are shown at the bottom of the figure, and episodes of thermal stratification are not observed.


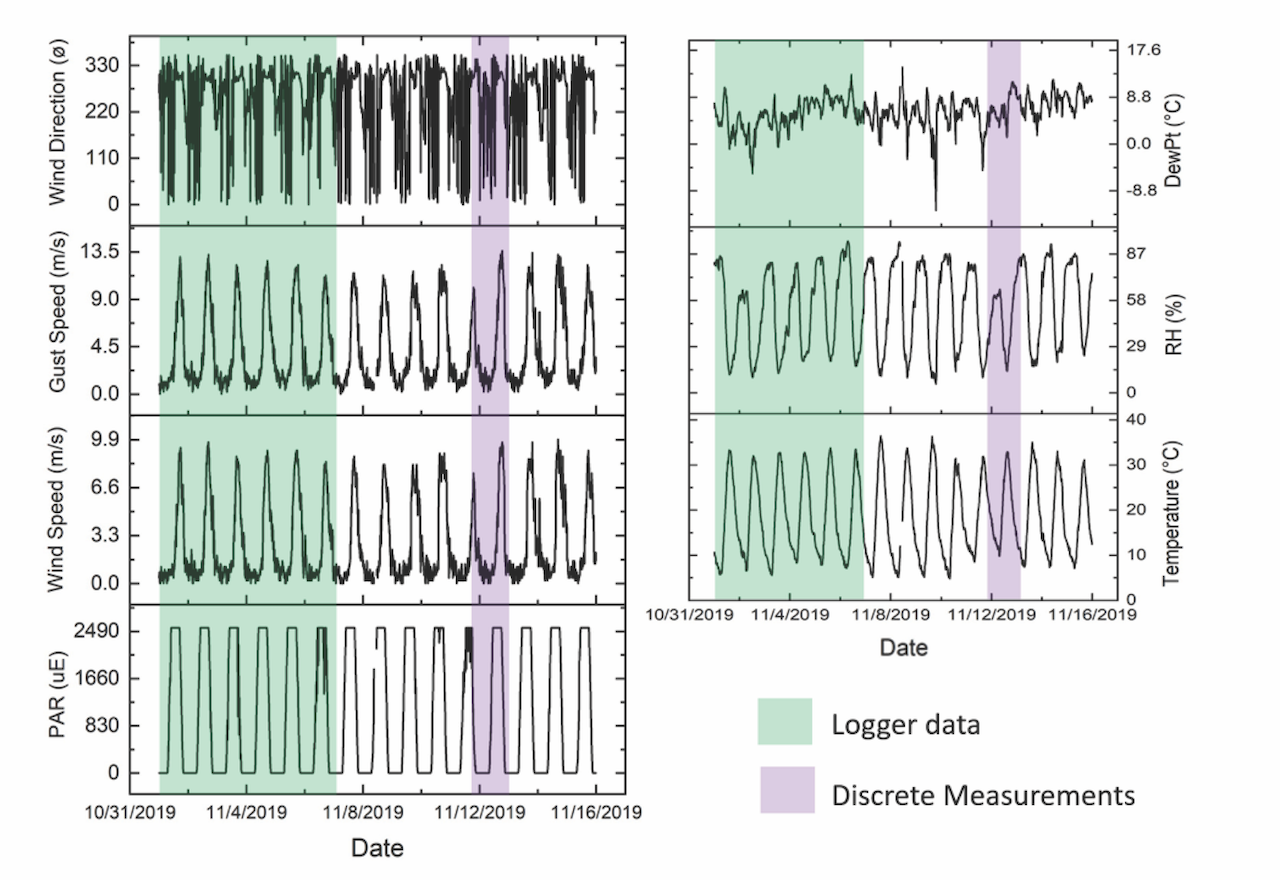


**S6 Fig. Local environmental conditions from meteorological station. Plots show local conditions of Photosynthetically Active Radiation (PAR), Wind Speed, Gust Speed, Temperature, Relative Humidity (RH), and Dew Point (DewPt) between November 1st to November 15th.** Purple box indicates the timing of the field campaign where discrete samples and in situ measurements collected using the Hanna 9829 were collected between November 12^th^ -13^th^, 2019. The green box indicates the time frame of logger data collection of surface and bottom lake water temperature.


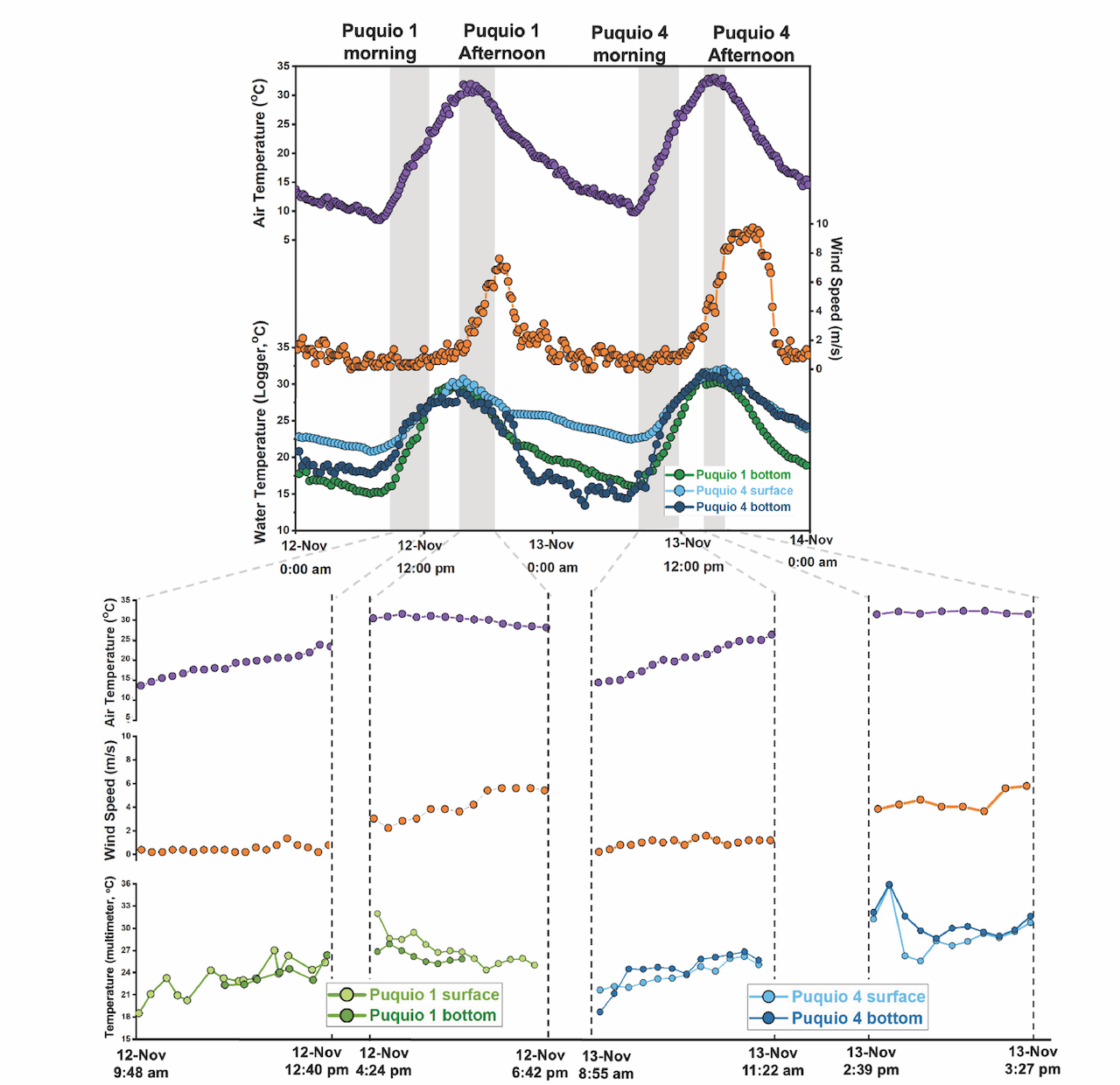


**S7 Fig. Comparison of meteorological station measurements and *in* situ measurements collected using the multimeter.** Top panel shows air temperature, wind speed, logger-based temperature measurements from Puquio 1 and 4 during the study period. Please note there are no surface measurements from this period because the logger malfunctioned. On the bottom panel, we compare the meteorological datasets (air temperature and wind speed) from the weather station to discrete measurements collected using the multimeter during the morning and afternoon field campaigns, the periods of which are highlighted in grey shaded boxes. Data gaps in Puquio 1 reflect shallow water depths (< 5 cm) that precluded dual measurements of surface and bottom water in this shallow, gently sloping lake.

**
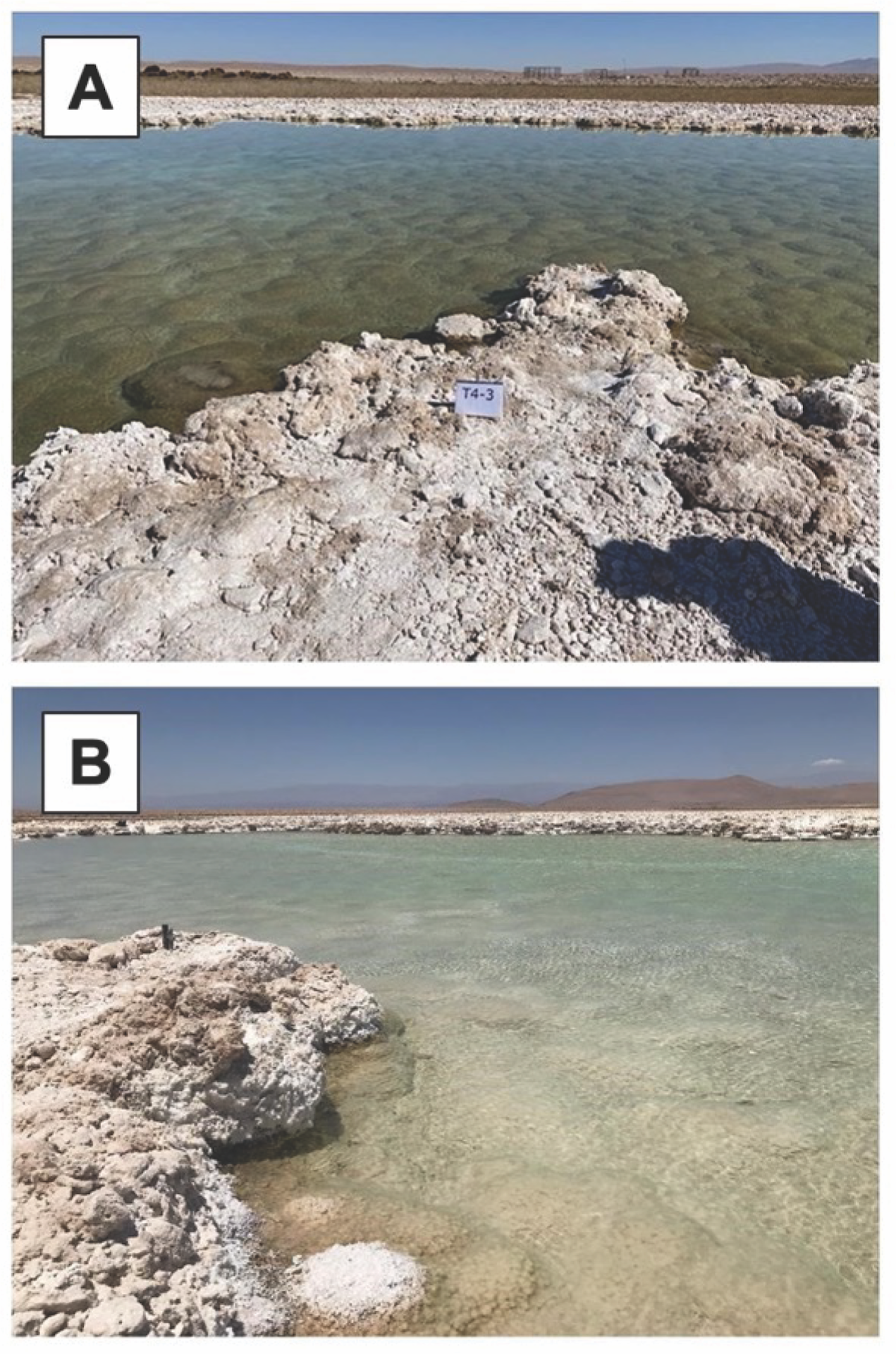
**

**S8 Fig. Field photographs (**a) Field photograph taken in the morning at Puquio 4 showing calm water surface without waves enabling clear view of lake substrate morphology. Sign with “T4-3” marks a measurement point. (b) Field photograph from the afternoon showing water surface with visible ripples in Puquio 4, which obscure view of lake substrate.


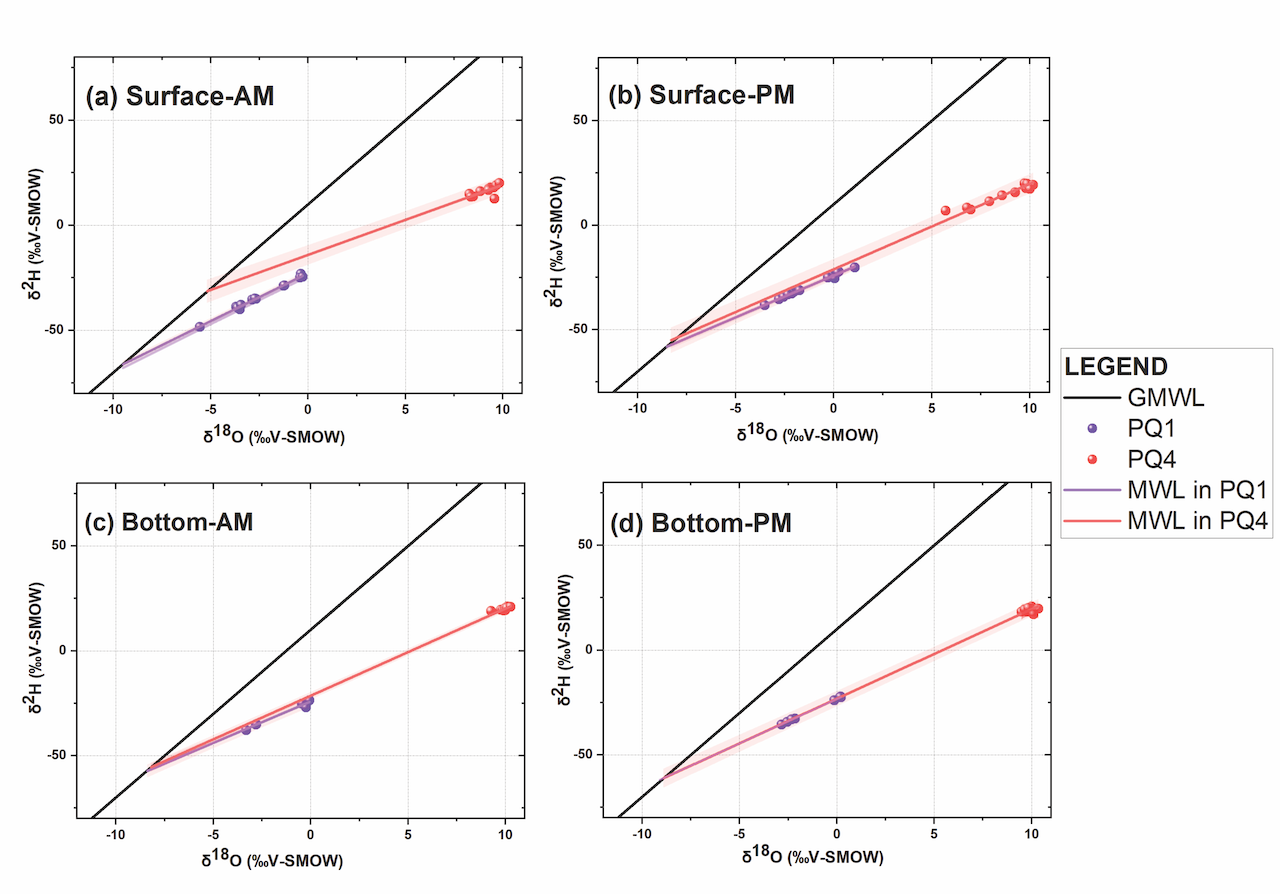


**S9 Fig. Cross plot of δ^2^H values (‰ V-SMOW) and δ^18^O values (‰ V-SMOW) for samples collected for lake waters from Puquio 1 (purple circles) and Puquio 4 (red circles)** in the (a) surface brines in the morning, (b) the surface brines in the afternoon, (c) the bottom brines in the morning, and (d) the bottom brines in the morning. The black line represents the global meteoric water line [75]. Transparent polygons are the 95% confidence interval.
